# Supplementary material for: Sporopollenin Capsules as Biomimetic Templates for the Synthesis of Hydroxyapatite and β-TCP
Source: Biomimetics (Basel). 2024 Mar 4;9(3):159. doi: 10.3390/biomimetics9030159 (PMC10968355; doi:10.3390/biomimetics9030159)
Supplement: Supplementary file 1 [file biomimetics-09-00159-s001.zip › biomimetics-2845702-supplementary.pdf]

Sporopollenin capsules as biomimetic templates for the synthesis of hydroxyapatite and calcium phosphate.

Arianna De Mori, Daniel Quizon, Hannah Dalton, Berzah Yavuzyegit, Guido Cerri, Milan Antonijevic, Marta Roldo

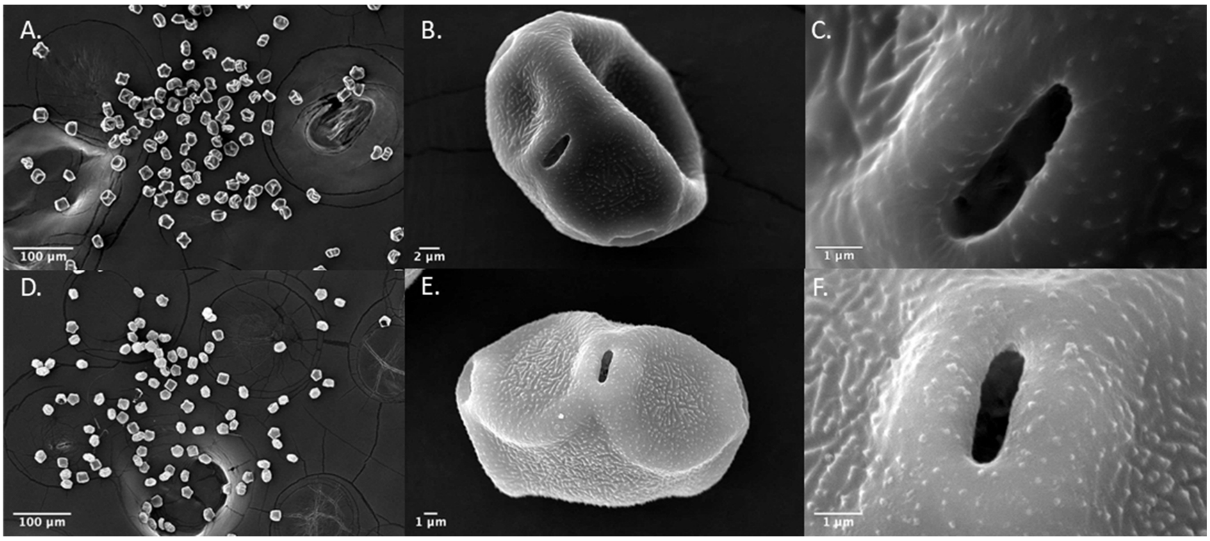

Figure S1. SEM images of alder pollen grains before (A-C) and after (D-F) acid washing.

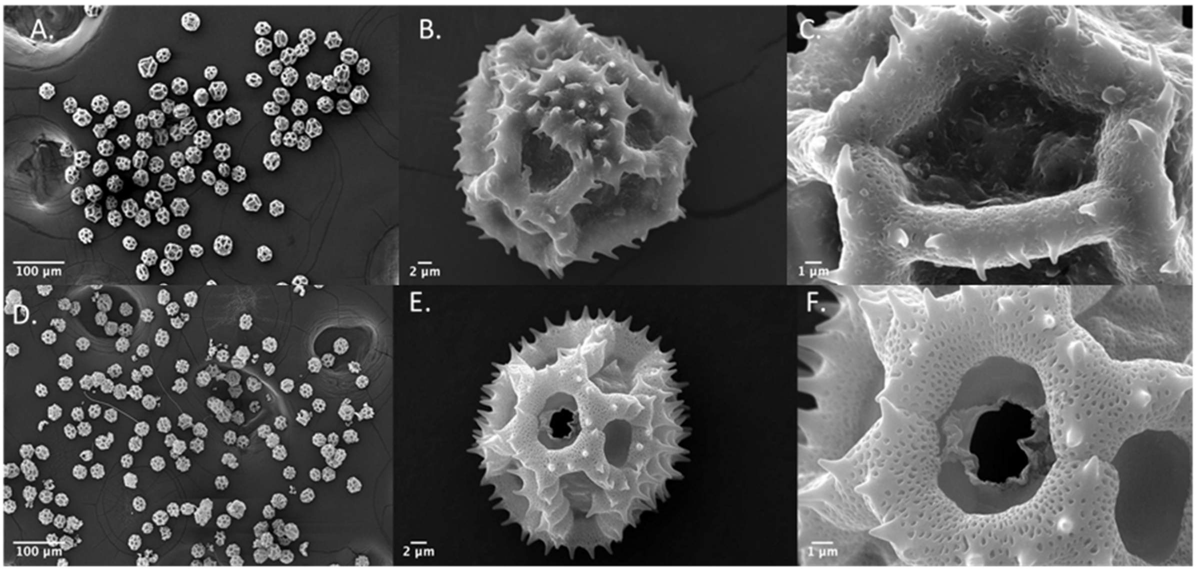

Figure S2. SEM images of dandelion pollen before (A-C) and after acid treatment (D-F).

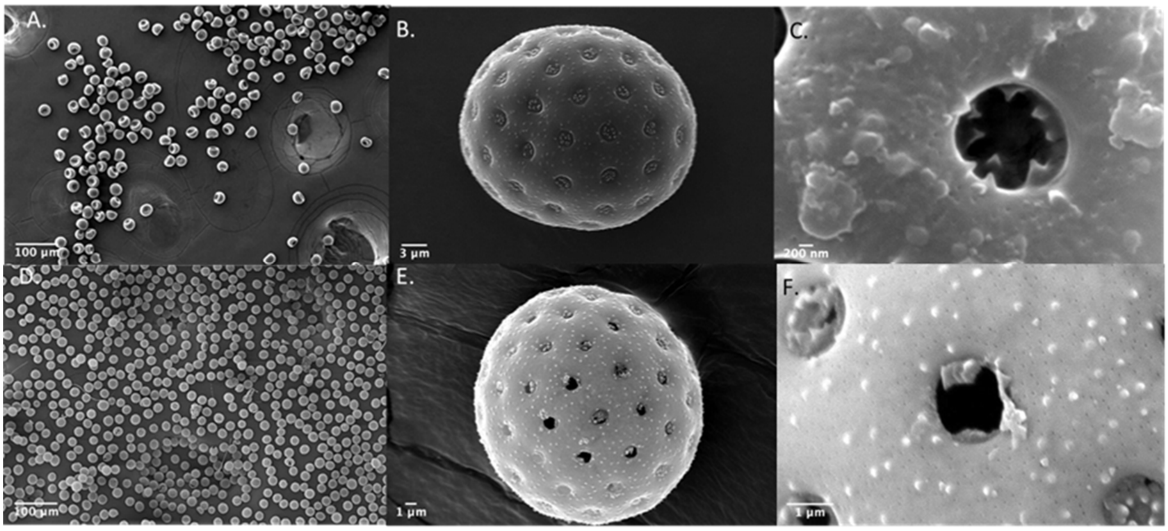

Figure S3. SEM images of lamb's quarters pollen before (A-C) and after acid washing (D-F).

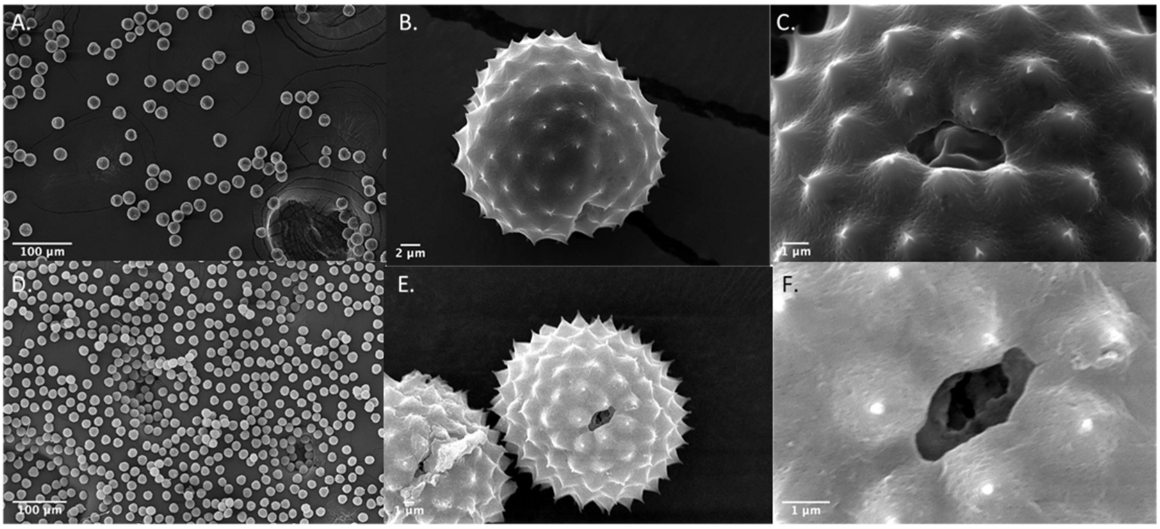

Figure S4. SEM photographs of ragweed pollen before (A-C) and after acid washing (D-F).

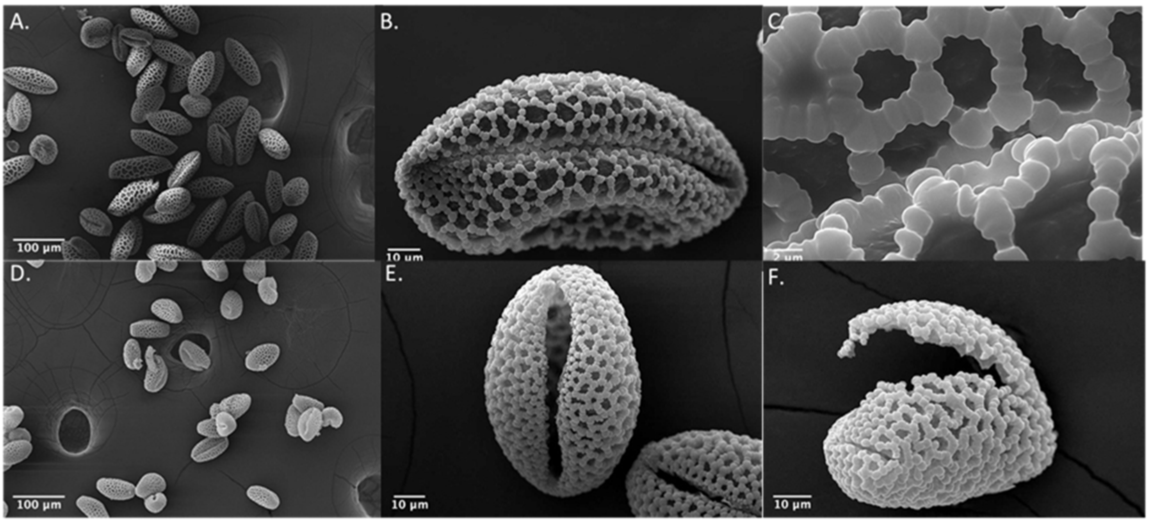

Figure S5. SEM photographs of stargazer lily grain before (A-C) and after (D-F) acid washing.

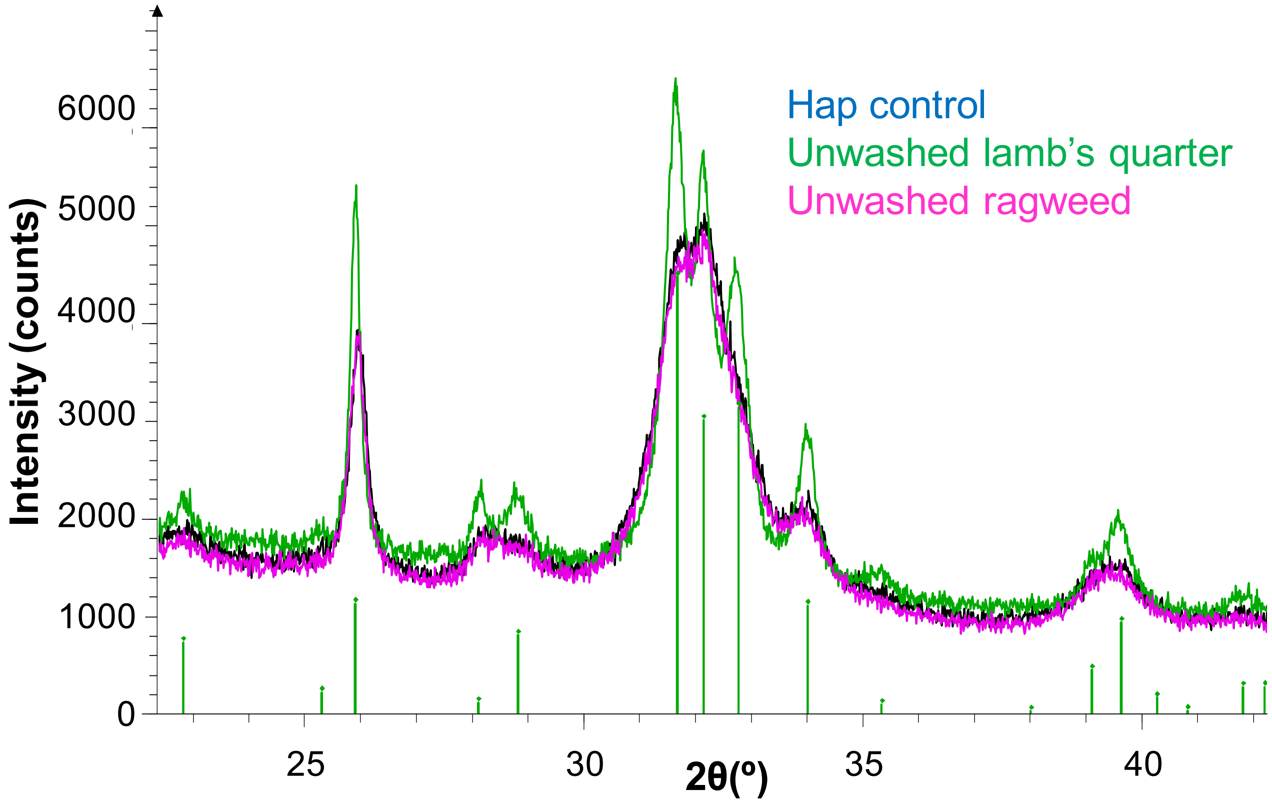

Figure S6 A. XRD patterns (2θ range 22.5-42.2°) of HAp control, unwashed lamb's quarter, and unwashed ragweed. Green bars: HAp (PDF N. 72-1243).

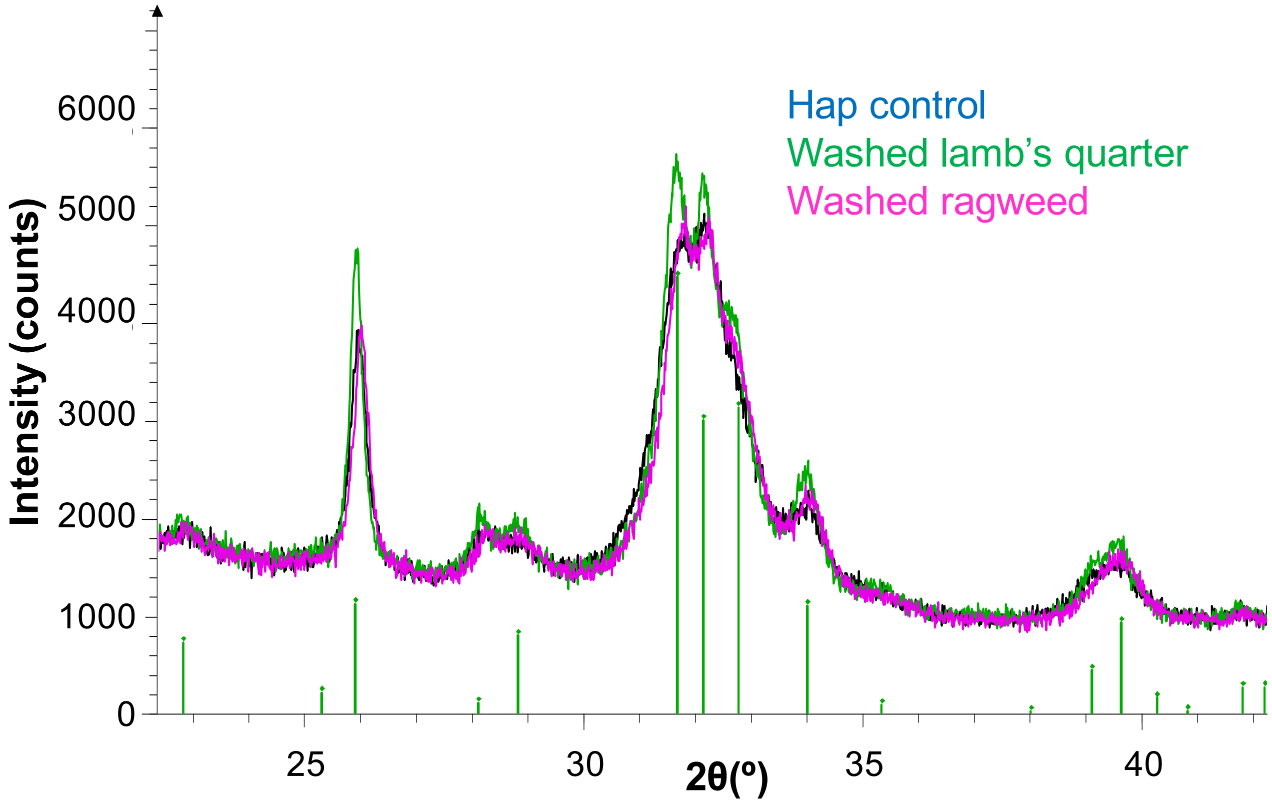

Figure S6 B. XRD patterns (2θ range 22.5-42.2°) of HAp control, washed lamb's quarter, and washed ragweed. Green bars: HAp (PDF N. 72-1243).

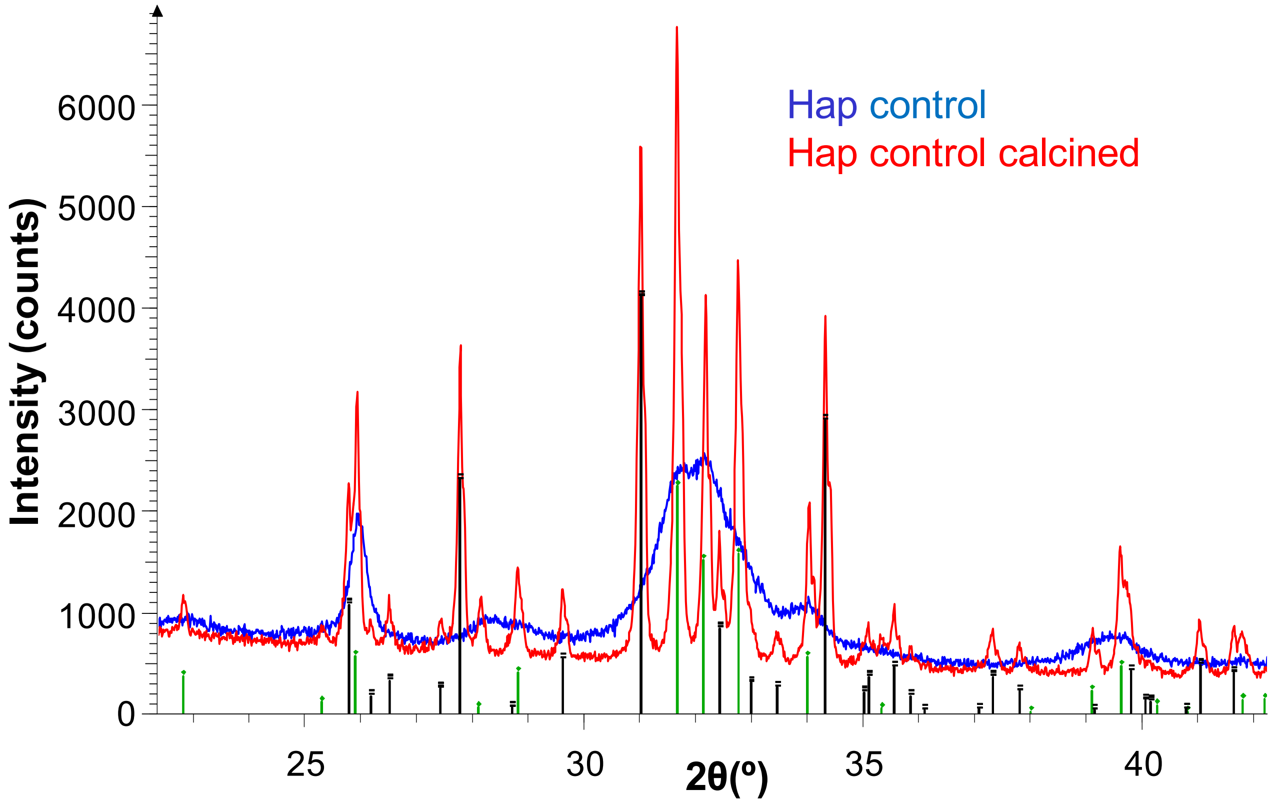

Figure S6 C. XRD patterns (2θ range 22.5-42.2°) of HAp control A) before and B) after calcination. Green bars: HAp (PDF N. 72-1243). Black bars: β-TCP (PDF N. 70-2065).

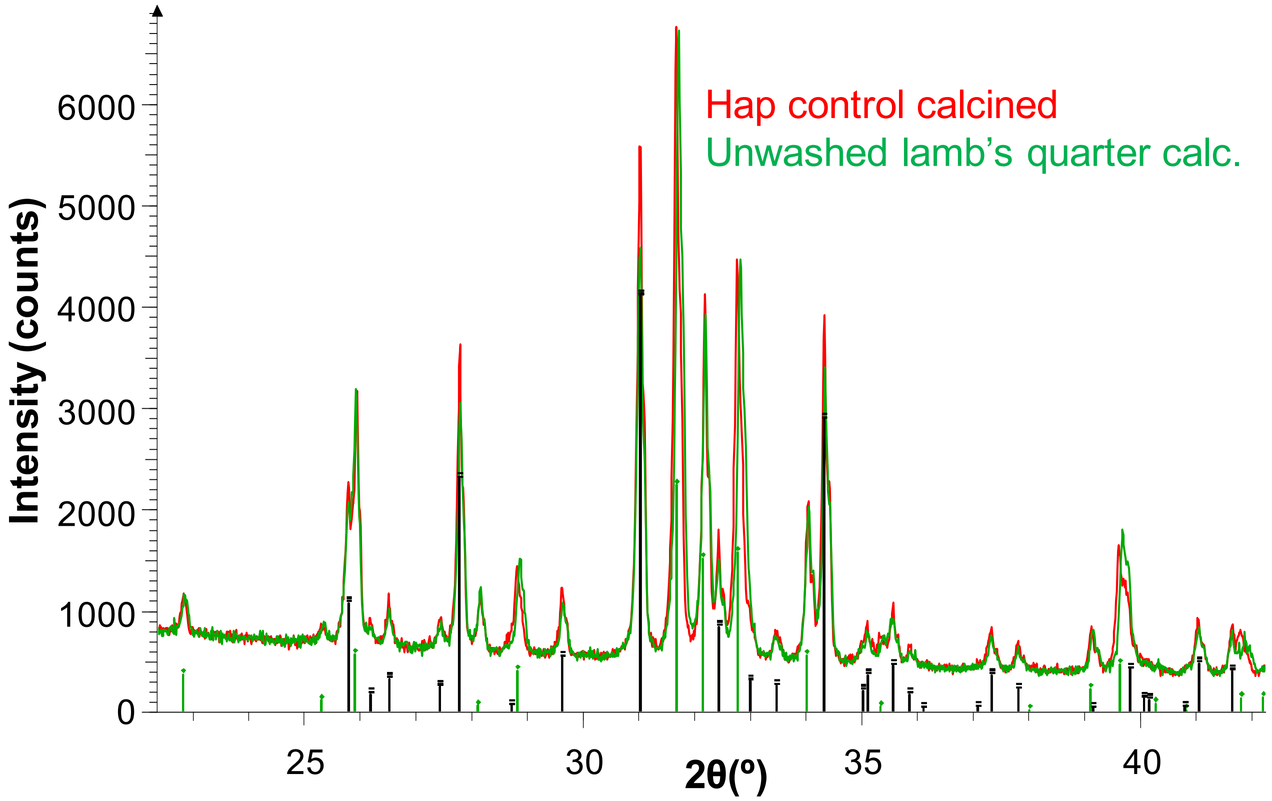

Figure S6 D. XRD patterns (2θ range 22.5-42.2°) of the HAp control and unwashed lamb's quarter samples after calcination. Green bars: HAp (PDF N. 72-1243). Black bars: β-TCP (PDF N. 70-2065).

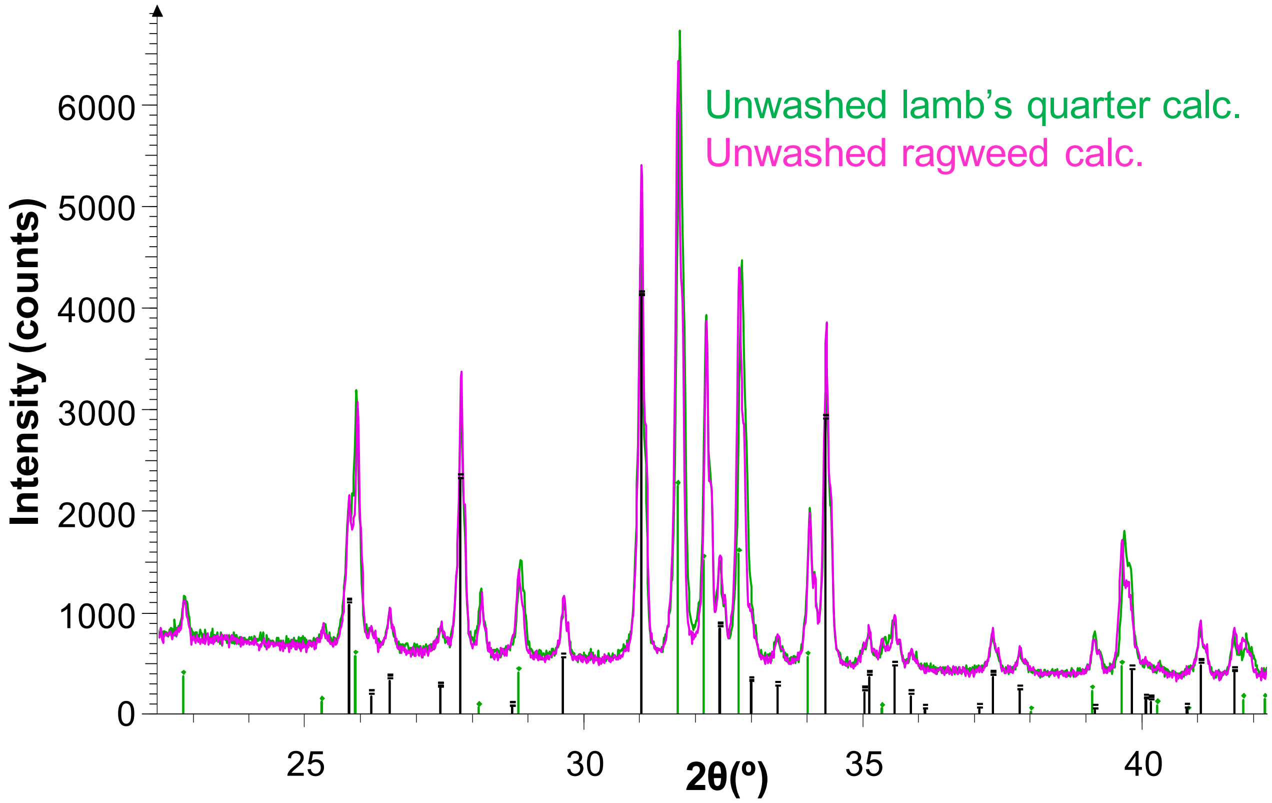

Figure S6 E. XRD patterns (2θ range 22.5-42.2°) of the unwashed lamb's quarter and ragweed samples after calcination. Green bars: HAp (PDF N. 72-1243). Black bars: β-TCP (PDF N. 70-2065).

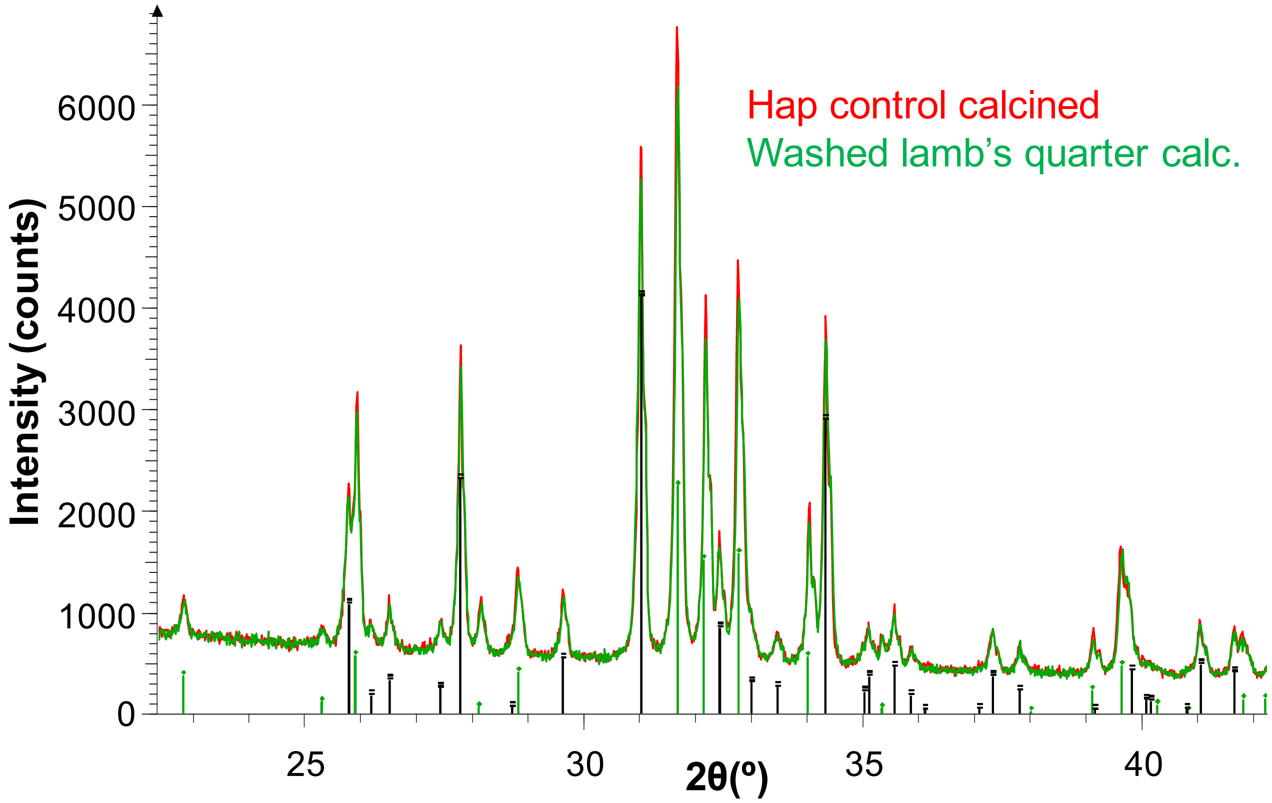

Figure S6 F. XRD patterns (2θ range 22.5-42.2°) of the HAp control and washed lamb's quarter samples after calcination. Green bars: HAp (PDF N. 72-1243). Black bars: β-TCP (PDF N. 70-2065).

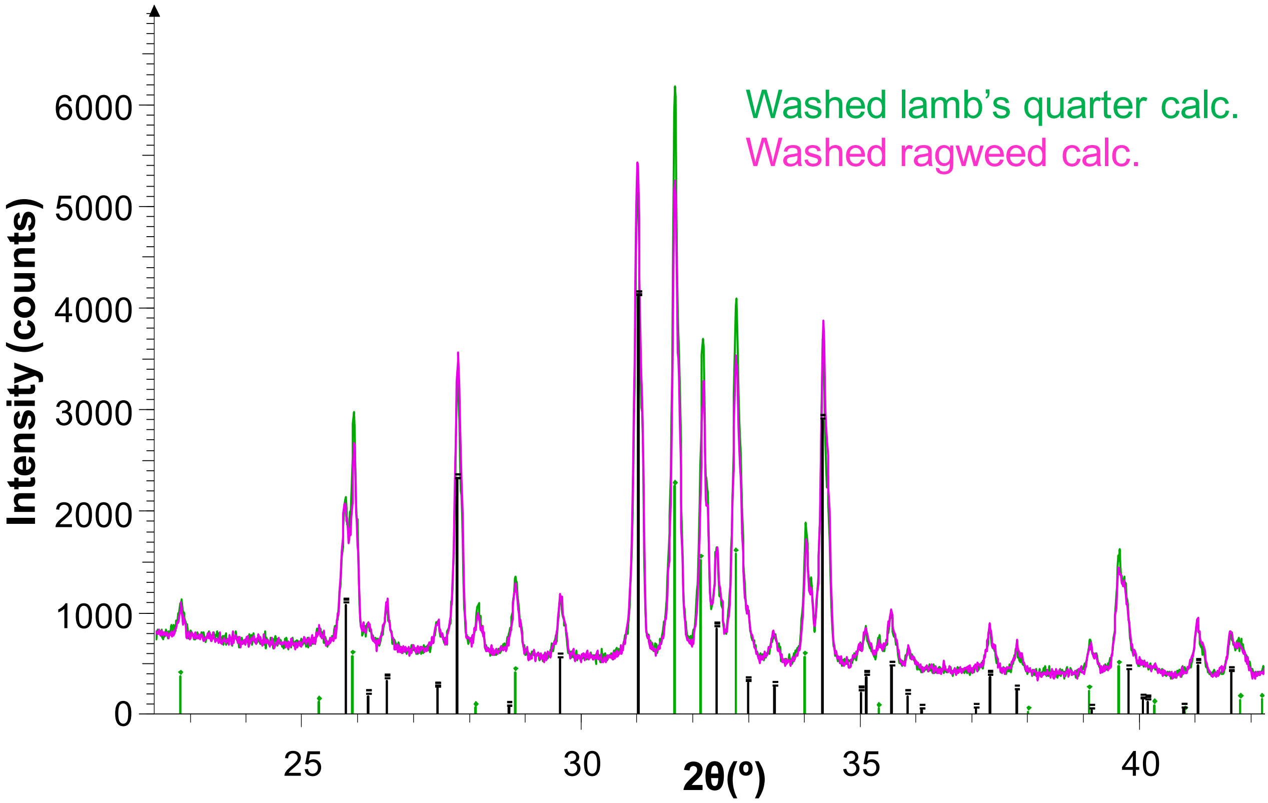

Figure S6 G. XRD patterns (2θ range 22.5-42.2°) of the washed lamb's quarter and ragweed samples after calcination. Green bars: HAp (PDF N. 72-1243). Black bars: β-TCP (PDF N. 70-2065).
